# Electrical properties and mechanical stability of anchoring groups for single-molecule electronics

**DOI:** 10.3762/bjnano.6.159

**Published:** 2015-07-17

**Authors:** Riccardo Frisenda, Simge Tarkuç, Elena Galán, Mickael L Perrin, Rienk Eelkema, Ferdinand C Grozema, Herre S J van der Zant

**Affiliations:** 1 Kavli Institute of Nanoscience, Delft University of Technology, Lorentzweg 1, 2628 CJ Delft, The Netherlands; 2 Department of Chemical Engineering, Delft University of Technology, Julianalaan 136, 2628 BL Delft, The Netherlands; 3 Current address: Arcelik A.S.Central R&D Department, 34950 Tuzla/Istanbul, Turkey

**Keywords:** anchoring groups, coherent transport, current–voltage, molecular electronics, single molecule

## Abstract

We report on an experimental investigation of transport through single molecules, trapped between two gold nano-electrodes fabricated with the mechanically controlled break junction (MCBJ) technique. The four molecules studied share the same core structure, namely oligo(phenylene ethynylene) (OPE3), while having different aurophilic anchoring groups: thiol (SAc), methyl sulfide (SMe), pyridyl (Py) and amine (NH_2_). The focus of this paper is on the combined characterization of the electrical and mechanical properties determined by the anchoring groups. From conductance histograms we find that thiol anchored molecules provide the highest conductance; a single-level model fit to current–voltage characteristics suggests that SAc groups exhibit a higher electronic coupling to the electrodes, together with better level alignment than the other three groups. An analysis of the mechanical stability, recording the lifetime in a self-breaking method, shows that Py and SAc yield the most stable junctions while SMe form short-lived junctions. Density functional theory combined with non-equlibrium Green’s function calculations help in elucidating the experimental findings.

## Introduction

Molecular-scale electronics is a field that in recent years experienced an enormous growth thanks to the development of reliable techniques to trap and electrically contact single molecules [1–3]. One such a technique involves the break-junction (BJ) methods; two widely used BJ methods are the mechanically controlled (MCBJ) and the scanning probe microscopy (SPMBJ) break junctions. These create and displace atomic-sized electrodes so that reproducibly single-molecule junctions are formed [4–7]. In the past years it has been shown that the electronic properties of single-molecule junctions depend both on the molecular core and on the interfaces between molecule and metal [8–21]. Especially important are the alignment of the frontier orbitals to the metal Fermi level and the hybridization of these orbitals with states in the metallic electrodes [22–25].

**Figure 1:**
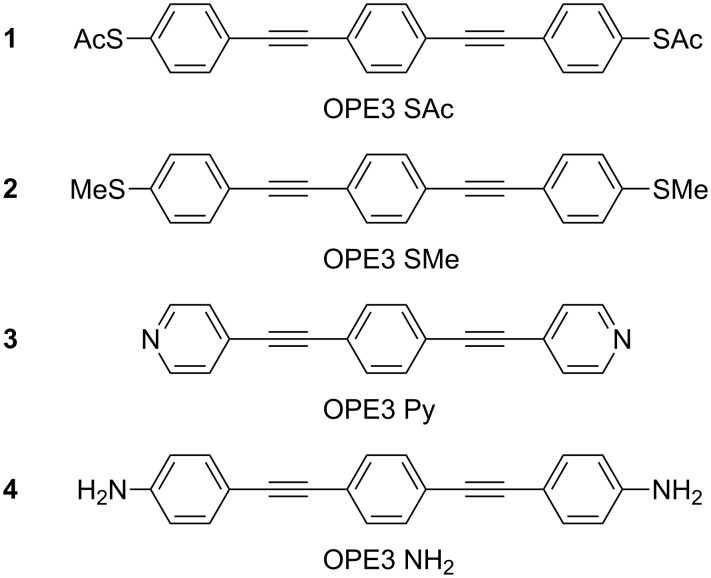
Chemical structures of the molecules studied in this work.

A mechanically stable contact and strong electronic coupling between the molecule and the metal electrode are essential to characterize charge transport in single-molecule junctions and to create new fundamental devices such as molecular motors or molecular machines [26–27]. Several previous studies have shown that stable and reproducible single-molecule junctions are formed if thiol (SH), methyl sulfide (SMe), pyridyl (Py) and amine (NH_2_), groups are used as anchoring groups [16,28–30]. In a comparative study Hong et al. [20], found that the low-bias conductance follows the trend SH *>* NH_2_*>* Py *>* CN by comparing a series of tolanes anchored to gold break-junction electrodes. Additionally, they report that the mechanical stability and the probability of forming a junction is highest for Py, followed by SH. Moreno-García et al. [15] recently compared a series of oligoyne molecular wires with Py, NH_2_, CN, SH and benzothiophene (BT) as anchoring groups. They find that the conductance is such that BT *>* SH *>* NH_2_*>* Py *>* CN, and the attenuation factor, β, that describes the length-dependence of the conductance, follows SH = Py *>* BT *>* NH_2_*>* CN. While SH and Py groups have been extensively studied and compared in single-molecule junctions, SMe groups have not been thoroughly characterized. Park et al. [16] have compared SMe to NH_2_ and dimethylphosphine (PMe_2_) and found that the conductance and the mechanical stability follow the sequence PMe_2_*>* SMe *>* NH_2_. In these previous studies the main focus was on the absolute magnitude of the conductance and its dependence on the length of the molecule. Tsutsui et al. [31], on the other hand, focused on the stability of SH and NH_2_ anchoring groups finding that SH exhibits a lifetime five orders of magnitude larger than that of NH_2_. Recently González et al. [32] studied NH_2_ anchored OPE3 molecules as a function of electrode speed at room temperature. The authors concluded that thermal breaking effects are not present and that the formed junctions lasted for more than ten seconds.In this paper we present a comprehensive study on the role of the anchoring groups both on the electronic and mechanical properties of single-molecule junctions formed with the MCBJ technique. In particular, we characterize room-temperature transport through four oligo(phenylene ethynylene) (OPE3) model compounds carrying different anchoring groups, thiol (**1**), methyl sulfide (**2**), pyridyl (**3**) and amine (**4**), reported in Figure 1, in order to explore how the nature of the molecule–electrode contact influences the conductance measurements. The electrical properties were studied by measuring the conductance and current–voltage characteristics as a function of electrode distance. A statistical analysis of the *I*–*V*s with a fit to a single-level model allows to quantify the electronic coupling between the various molecules and the electrodes and the injection barrier [33]. Additionally, we have performed self-breaking measurements, in which we measure the low-bias conductance of a molecular junction as a function of time until the spontaneous rupture of the junction occurs. These measurements give information about the mechanical stability of the different anchoring groups. Together, these experiments give a detailed insight in the electronic and mechanical characteristics of different anchoring groups and can be used as guidance when designing functional single-molecule devices.

## Experimental

**Chemicals and Synthesis.** All chemicals and solvents were purchased from commercial sources. OPE3-SAc (**1**), with acetyl-protected thiol groups (SAc), was purchased from Sigma-Aldrich. Details of synthesis and characterization of OPE3-SMe (**2**), OPE3-Py (**3**) and OPE3-NH_2_ (**4**) are summarized in Supporting Information File 1.

**Figure 2:**
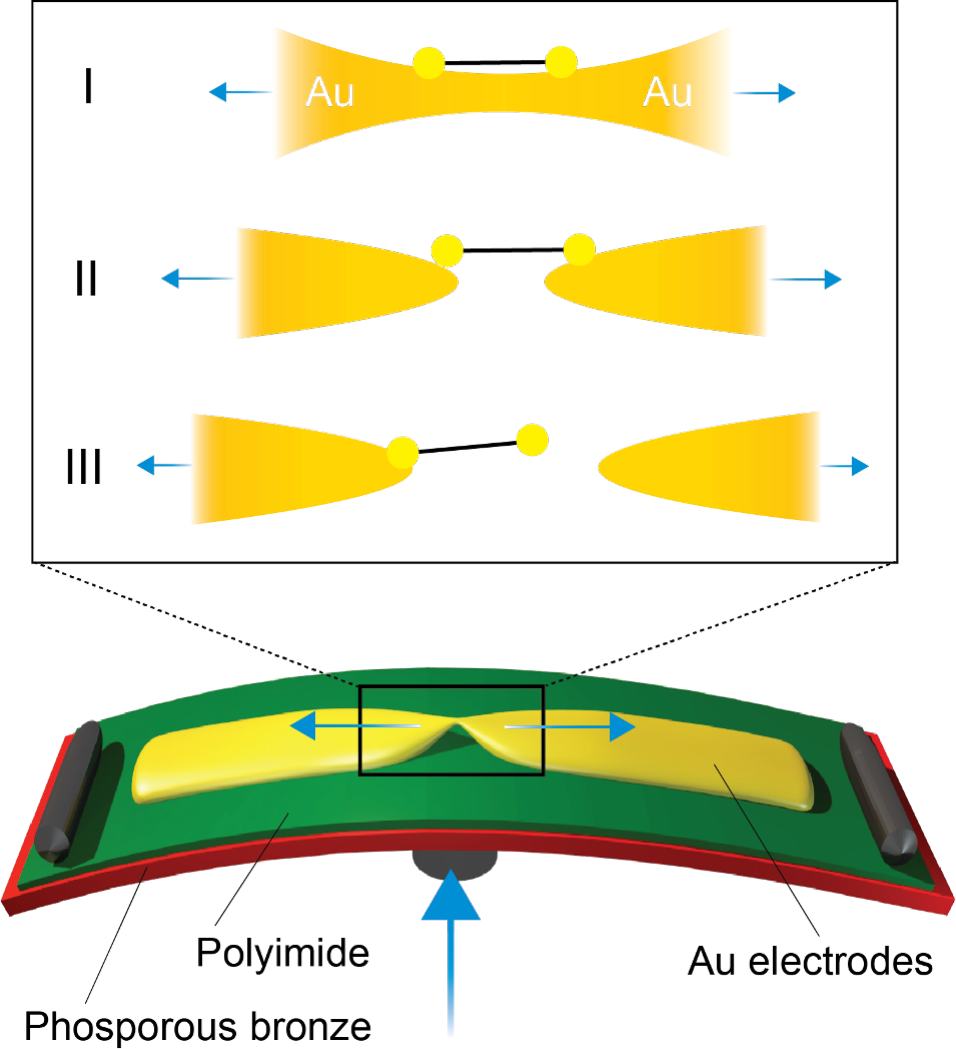
Drawing of the MCBJ setup and schematics of the formation of a molecular junction.

**Sample preparation.** Figure 2 presents a schematics of an MCBJ sample used to measure single-molecule junctions. Sample fabrication, published elsewhere [34–35], is briefly described in the following: the flexible substrate consists of a fine-polished, 500 μm thick, phosphorous bronze sheet coated with 6 μm of insulating polyimide. A gold wire, with a 50 nm wide constriction in the middle, is fabricated on top of the polyimide with standard electron beam lithography, evaporation and lift-off techniques. After a final O_2_/CF_4_ plasma etching step, performed to suspend the central constriction, the sample is mounted in a three-point bending mechanism and electrically contacted. By bending the substrate with a pushing rod controlled by a stepper motor or a piezoelectric element, the gold wire can be broken thereby forming two tip-shaped electrodes. The vertical displacement of the pushing rod translates in an horizontal displacement in the plane of the electrode with an attenuation factor of 5 · 10^−5^. Molecules **1**–**4** were deposited onto the MCBJ device by drop-casting a solution (2 μL of 1 mM in dichloromethane) on the freshly prepared gold nano-electrode. Just before the deposition of molecule **1**, we added to the molecular solution two equivalents, relative to the number of OPE3 SAc molecules, of tetrabutylammonium hydroxide (TBAH) dissolved in dichloromethane, i.e., one TBAH molecule per SAc group. The addition of TBAH is known to deprotect the acetyl-protected thiol groups and promote the formation sulfur-gold bonds [36–37].

**Transport measurements.** The study of molecular transport has been conducted in air and at room temperature, using the MCBJ gold nano-electrodes to connect individual molecules. In the first experiment we break and reform the gold contact while recording the current with an applied bias voltage of typically 0.1 V separating the electrodes at a speed of 5 nm/s. A feedback on the conductance is used: we break for 6 nm after reaching 20*G*_0_ and we close the junction until the conductance reaches 40*G*_0_, with *G*_0_ = 2e^2^/*h* being the quantum of conductance. Typically, we measured more than 2000 individual conductance-distance breaking traces for each molecule; these traces are used to construct the one- and two-dimensional histograms.

To measure current–voltage characteristics of single-molecule junctions we separate the electrode at a speed of 0.01 nm/s. While the gold wire is still intact, we sweep the bias between −0.1 V and 0.1 V ramped at a speed of 0.3 V/s. When the conductance, measured at 0.1 V of bias, reaches a value smaller than 0.1*G*_0_ we start to sweep the voltage between −0.7 V and 0.7 V recording 100 points per *I*–*V* at a rate of 0.05 V/s. Once the current drops below 0.1 nA over the whole bias voltage range we reform the gold contact until the conductance reaches 40*G*_0_ and a new breaking trace starts. In this way we recorded between 300 and 500 individual *I*–*V* breaking traces for each molecule that yield between 3000 and 5000 individual *I*–*V*s. In the third type of experiments we stretch the gold wire until the low-bias conductance reaches 10*G*_0_. We then apply a bias voltage of 0.1 V while recording the current. Due to the strain in the Au wire, it spontaneously breaks (self-breaking) and the conductance at 0.1 V is measured as a function of time. Once the conductance falls below 2 · 10^−7^*G*_0_ for more than 1 minute we reform the junction, approaching the two gold electrodes until the conductance reaches 40*G*_0_. The sequence is then repeated. For each molecule we record between 100 and 300 of such conductance-time breaking traces.

**Theoretical calculations.** Electronic ground state properties are calculated using density functional theory (DFT) as implemented in the ADF package [38–39], using the GGA-PBE functional [40], and the triple-ζ plus polarization (TZP) basis set. The zeroth order regular approximation (ZORA) to the Dirac equation was used to account for relativistic effects in the electrodes. Each molecule is connected to two pyramidal gold electrodes consisting of 55 atoms each and initially separated by a gap of 0.7 nm (center-to-center distance between the two gold adatoms is 0.94 nm). The geometry was converged to energy changes of less than 10^−3^ hartree, energy gradients of less than 10^−3^ hartree/Å maximum and 6.7 · 10^−4^ hartree/Å RMS. We then start to separate the gold electrodes in steps of 4 pm and for each new gap size we relax the geometry of the molecule and of the two outer gold layers. Every ten steps (40 pm) we calculate the transmission through the molecular junction using non-equilibrium Green’s function (NEGF) formalism by connecting the outer gold layer to wide-band limit electrodes. To account for well-known problems in the DFT eigenvalues we include DFT + Σ corrections [41].

## Results and Discussion

**Figure 3:**
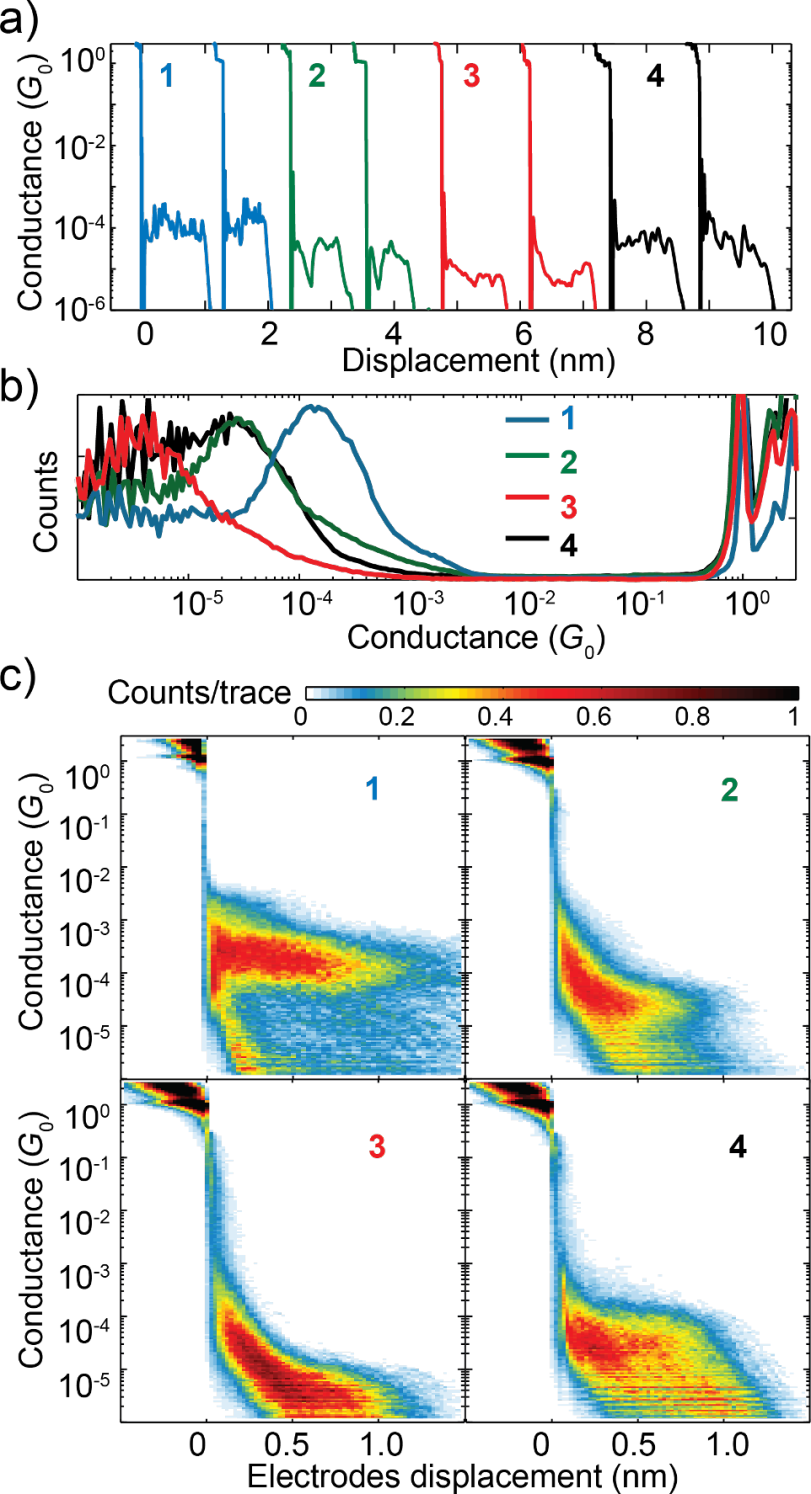
(a) Individual breaking traces in presence of the molecules **1**–**4**. The applied bias is 0.1 V and the electrode retraction speed is 5 nm/s. The traces are offset along the x-axis for clarity. (b) Conductance histograms built from more than 2000 breaking traces recorded in presence of the molecules **1**–**4**. The breaking traces have been logarithmically binned, in the conductance axis, with 30 bins/decade. (c) Conductance–distance histograms built from the breaking traces of molecules **1**–**4**. The displacement axis has been binned with 45 bins/nm.

Figure 3a shows examples of conductance-distance breaking traces recorded in presence of molecules **1**–**4** and plotted on a semi-logarithmic scale for the conductance. The traces show step-like features for conductance values above 1*G*_0_, where the current flows through the gold constriction, and the presence of a final plateau at the value of 1*G*_0_ indicates the formation of a single gold atom junction, a sign of atomically sharp electrodes. Between 1*G*_0_ and 10^−3^*G*_0_ a sharp drop of a few orders of magnitude in conductance is visible, that is attributed to the stress-releasing jump-out-of contact of the gold adatoms accompanied by the instantaneous formation of a few Å gap between the now separated gold electrodes. In the region below 1*G*_0_ the successful formation of molecular junctions is evidenced by the appearance of conductance plateaus that extend for a displacement of about 1 nm.

To perform a statistical analysis, we record more than 2000 individual breaking traces for each molecule. Figure 3b presents the one-dimensional conductance histogram of each molecule, built from the individual breaking traces by logarithmically binning the conductance axis. The histograms show regions of high counts above 1*G*_0_, due to stable atomic configurations of the gold electrodes. In the sub-*G*_0_ region, the most probable conductance value of each molecule is extracted from the peaks in the histograms, fitted by a log-normal distribution. In this distribution, the logarithm of the random variable is normally distributed and the two fit parameters are μ, the location parameter, and σ, the scale parameter, respectively related to the mean and the geometric standard deviation of the normal distribution. The parameters extracted from the fit are listed in Table 1. We extract the following values for the most probable conductance (defined as the mode of the distribution): SAc = 2.8 · 10^−4^*G*_0_, SMe = 5.9 · 10^−5^*G*_0_, Py = 2.2 · 10^−6^*G*_0_ and NH_2_ = 7.0 · 10^−5^*G*_0_. The difference in conductance observed for the thiol and the methyl sulfide junctions indicates that the methyl groups are not cleaved away when the molecules make contact with the gold contacts [42].

**Table 1:** Fitting parameters extracted from the fit of the conductance peaks to a log-normal distribution.

Molecule	μ	σ

**1**	−7.5	0.84
**2**	−9.0	0.86
**3**	−10.1	0.76
**4**	−9.0	0.77

Since the absolute value of displacement, at which the gold wire ruptures, changes from trace to trace, we define the zero of displacement, for each breaking trace, as the point where the conductance drops below 0.5*G*_0_. In this way, we can build two-dimensional conductance–displacement histograms by binning at the same time the conductance, with logarithmic bins, and the displacement, with linear bins. Figure 3c shows the two-dimensional conductance–length histograms of the four molecules investigated, where the color indicates the probability for a value of conductance to appear at a certain displacement during the stretch of the junction. Since the histograms are built without data selection they contain both breaking traces measured on a molecular junction, showing a characteristic plateau in conductance, and traces where no molecule was trapped between the electrode, showing an exponential decay of the conductance. The molecular junctions show up as broad, flat high-count regions that extend for around 1 nm; these indicate the most probable value for the single-molecule junction conductance. From the two-dimensional histograms one can see that the junctions formed with the four OPE3 molecules show different conductance values, depending on the anchoring group, and a length between 0.8 nm and 1.0 nm. The length of the four molecules, from sulfur to sulfur or from nitrogen to nitrogen, computed with DFT, falls between 1.7 nm and 2.0 nm. The observed displacement is smaller than the DFT values and should be corrected to include the instantaneous gap formation in gold break junctions estimated in literature to be close to 0.5 nm [43]. The length obtained by adding this value to the experimental total displacement suggests that we can stretch the single molecules between the MCBJ gold nano-contacts.

**Figure 4:**
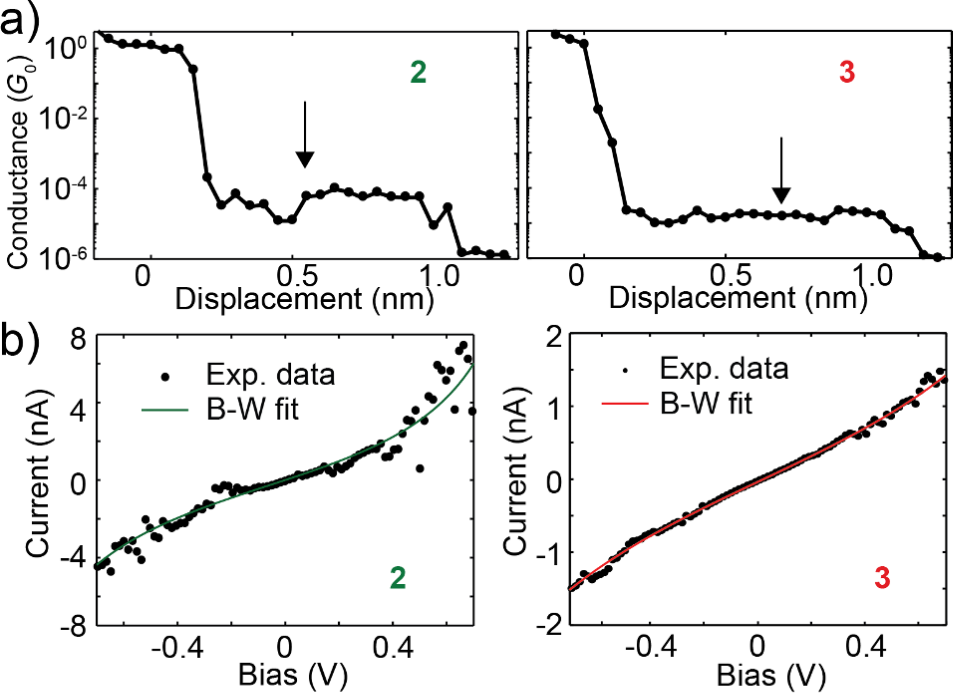
(a) Conductance versus displacement traces of the molecules **2** and **3**. The conductance is extracted from a linear fit of the current at low bias of each *I*–*V* measured during the stretching oh the molecular junction. (b) Two *I*–*V* curves, measured in the points indicated by the arrows in (a). The solid line corresponds to a single-level model fit to the data, the parameters of the fit are: **2** (ε_0_ = 0.55 eV, Γ = 4 meV) and **3** (ε_0_ = 0.71 eV, Γ = 2 meV).

To better understand the origin of the difference in conductance between the four different anchored molecules we have measured *I*–*V*s characteristics of the individual molecular junctions as a function of the electrodes displacement. Before discussing the *I*–*V*s, we show in Figure 4a the conductance, extracted from the *I*–*V*s, as a function of the displacement for **2** and **3**. Each dot represents the conductance obtained by linearly fitting the *I*–*V* characteristic between −0.1 V and 0.1 V. The breaking traces show the quantized conductance around 1*G*_0_, and conductance plateaus below 10^−4^*G*_0_, at similar values as shown in Figure 3.

Figure 4b presents two *I*–*V*s measured at the points indicated by the arrows in Figure 4a; the dots indicate the experimental data while the solid lines are fits to the single-level model. From the two *I*–*V*s one can see that the current flowing in the junctions is larger for **2** than for **3** and that **2** shows a more non-linear curve. To extract quantitative information about the molecular junctions from the *I*–*V*s we fit the current to the asymmetric Breit–Wigner single-level model [19,33]. This model is useful to model the current flowing in molecular junctions as a function of three parameters: the electronic coupling between molecule and left and right electrode, respectively Γ_L_ and Γ_R_, and the injection barrier ε_0_, that is the misalignment between the Fermi energy of the electrodes *E*_F_ and the energy of the closest frontier orbital in the molecule ε_0_ = |*E*_F_−ε|, where ε is the energy of the molecular level. In the Landauer transport formalism [44], at zero temperature, the current is given by:

[1]
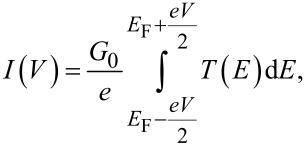


where *T*(*E*) is the transmission, that in the single-level model is a Lorentzian peak centered at ε_0_ and broadened by the electronic coupling Γ = Γ_L_ + Γ_R_:

[2]
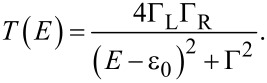


For off-resonant transport and at low-bias, *eV <<* ε_0_, the conductance is proportional to


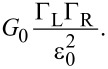


The fits in Figure 4 show a higher coupling and a level closer to the Fermi energy of the electrodes in the case of **2**. This translates in a higher conductance at low bias in accordance with the observations made for Figure 3 [45].

**Figure 5:**
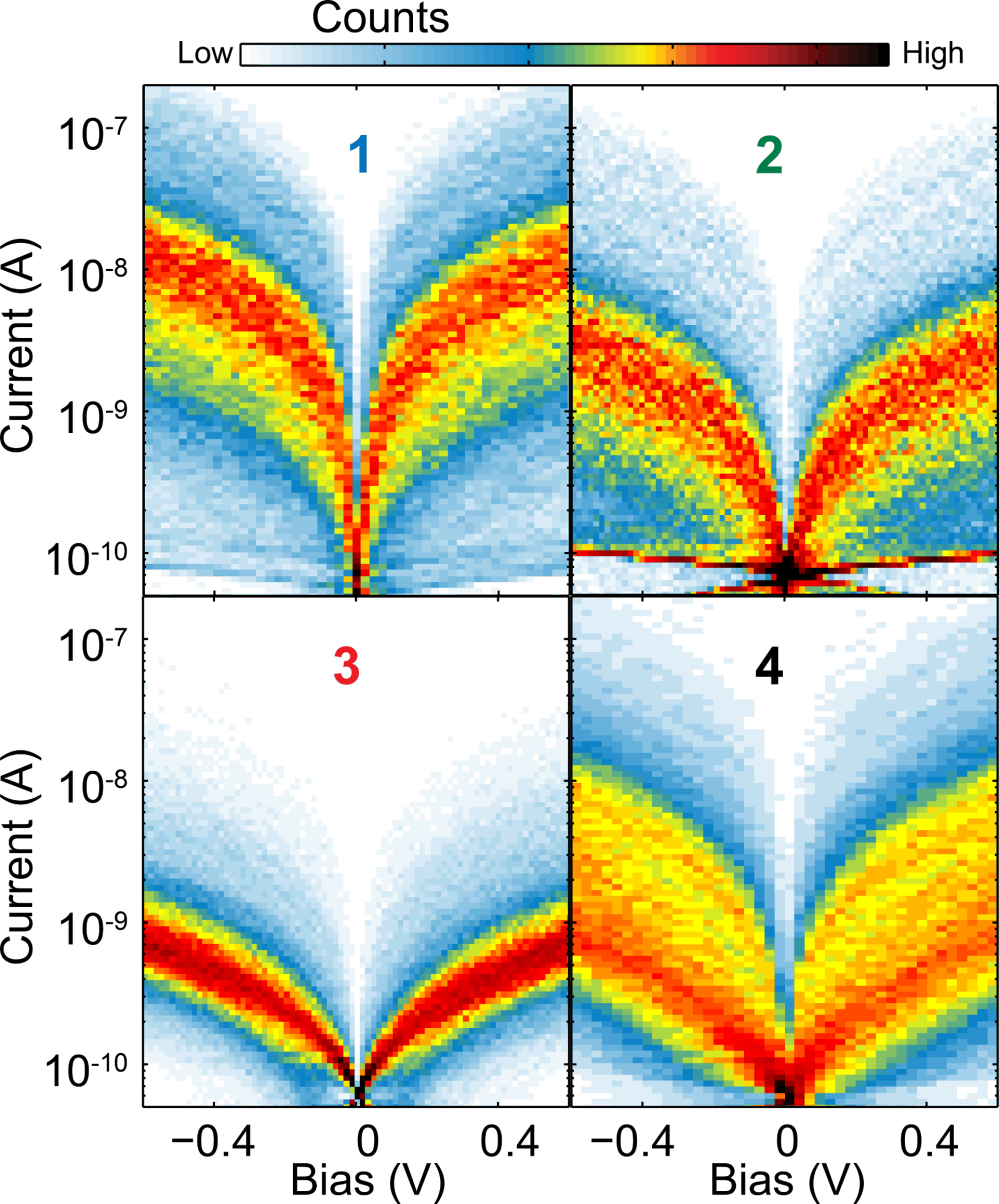
Two-dimensional color map plots of all the *I*–*V*s measured on molecules **1**–**4**. The bias axis is linearly binned with 40 bins/V and the current is logarithmically binned with 25 bins/decade.

We have measured hundreds of different junction breaking traces to perform a statistical analysis of the *I*–*V* characteristics. Figure 5 shows two-dimensional histograms, plotted in semi-logarithmic scale, built from all the *I*–*V*s measured while stretching the junctions in presence of molecules **1**–**4**. First, we note that each molecule shows a high-count region (in red), symmetric around zero bias; the current is the highest in **1**, followed by **2** and **4**, and last **3**, again in accordance with the conductance-length measurements reported in Figure 3.

**Figure 6:**
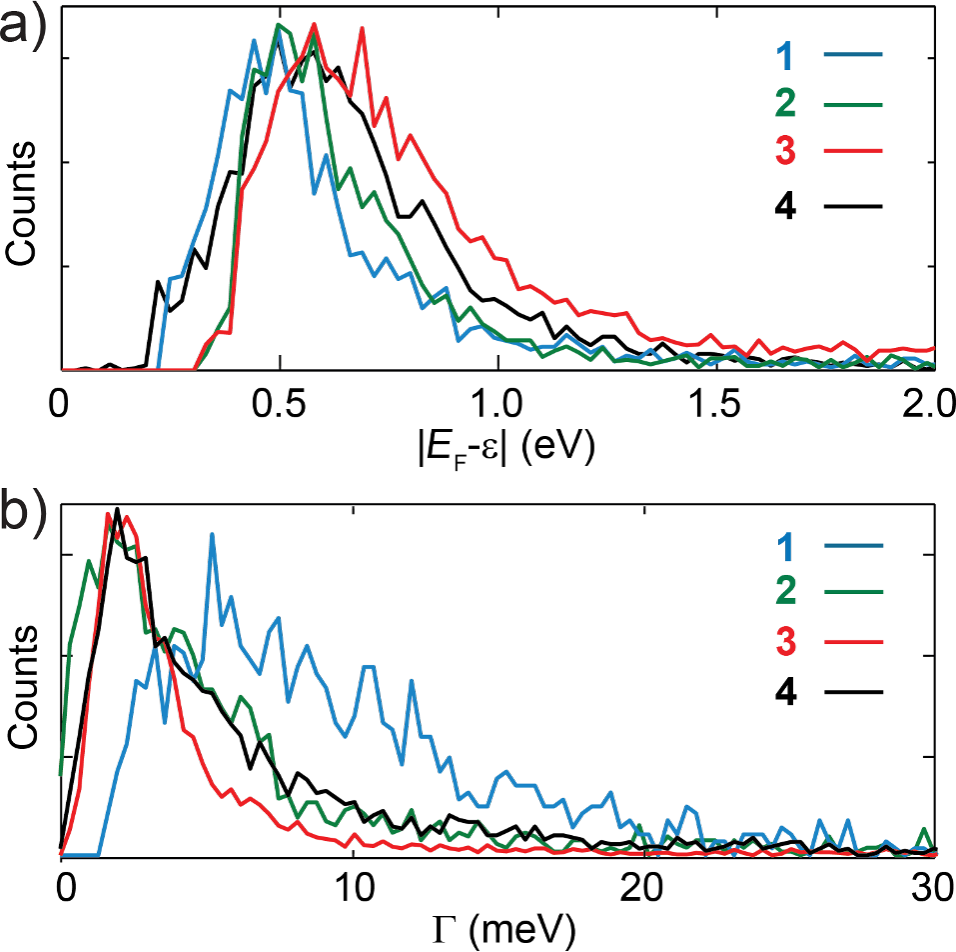
(a) One dimensional histograms of the injection barrier, for molecules **1**–**4**, extracted from the single level model (linearly binned with 36 bins/eV). (b) One dimensional histograms of the electronic coupling linearly binned with 3 bins/meV.

We fit each *I*–*V* characteristic to the single-level model, expressed in Equation 1, and extract the parameters ε_0_ and Γ. Figure 6 shows the linearly binned histograms of the fit parameters, ε_0_ and Γ, for the four molecules. The four histograms of the injection barrier in Figure 6a show an asymmetric peak, with a tail at larger energies. To quantify injection barrier and coupling we fit each parameter distribution to a log-normal distribution, as shown in Section 3 of Supporting Information File 1. We calculate subsequently the geometric mean of each distribution corresponding in a log-normal both to the median and to the 50th percentile. Table 2 reports the results for the average injection barrier and electronic coupling, the confidence intervals given correspond to the half width at half maximum of the different distributions. From this table we conclude that thiol groups give the highest coupling to the gold electrodes and the closest energy alignment. Methyl sulfide anchoring groups are characterized by a value for the injection barrier which is slightly higher than for the thiol case and a coupling which is a factor two smaller. Amine groups show an electronic coupling slightly smaller than the methyl sulfide case but a higher value for the injection barrier. The pyridyl anchored molecules have the largest values of the injection barrier and a coupling comparable to the methyl sulfide case.

**Table 2:** Single-level model parameters extracted from the fit to the experimental *I*–*V*s.

Molecule	ε_0_ (eV)	Γ (meV)

**1**	0.50 ± 0.15	8.4 ± 4.2
**2**	0.55 ± 0.14	4.3 ± 2.5
**3**	0.68 ± 0.22	2.8 ± 1.0
**4**	0.63 ± 0.18	4.1 ± 2.2

The analysis of the *I*–*V*s, fitted to the Breit–Wigner single-level model, suggests that the thiol groups are electronically strongly coupled to the electrodes and give the lowest injection barrier, corresponding to the best level alignment with the Fermi energy of the gold nano-electrodes. Interestingly, the injection barrier values that we find, between 0.5 eV and 0.7 eV, are comparable with charge injection barrier values, found in literature from photoemission spectroscopy of comparable OPE molecules [46–47]. In order to understand in more detail the transport in the single-molecule junctions mediated by the four different anchoring groups we performed DFT+Σ calculations combined with NEGF transport calculations, shown in Figure S6 of Supporting Information File 1. We find that the theoretical values of the conductance and of the electronic coupling compare well with the experimental values found for SAc, SMe and Py. In the case of NH_2_, both quantities are larger in the calculations than in the experiment.

**Figure 7:**
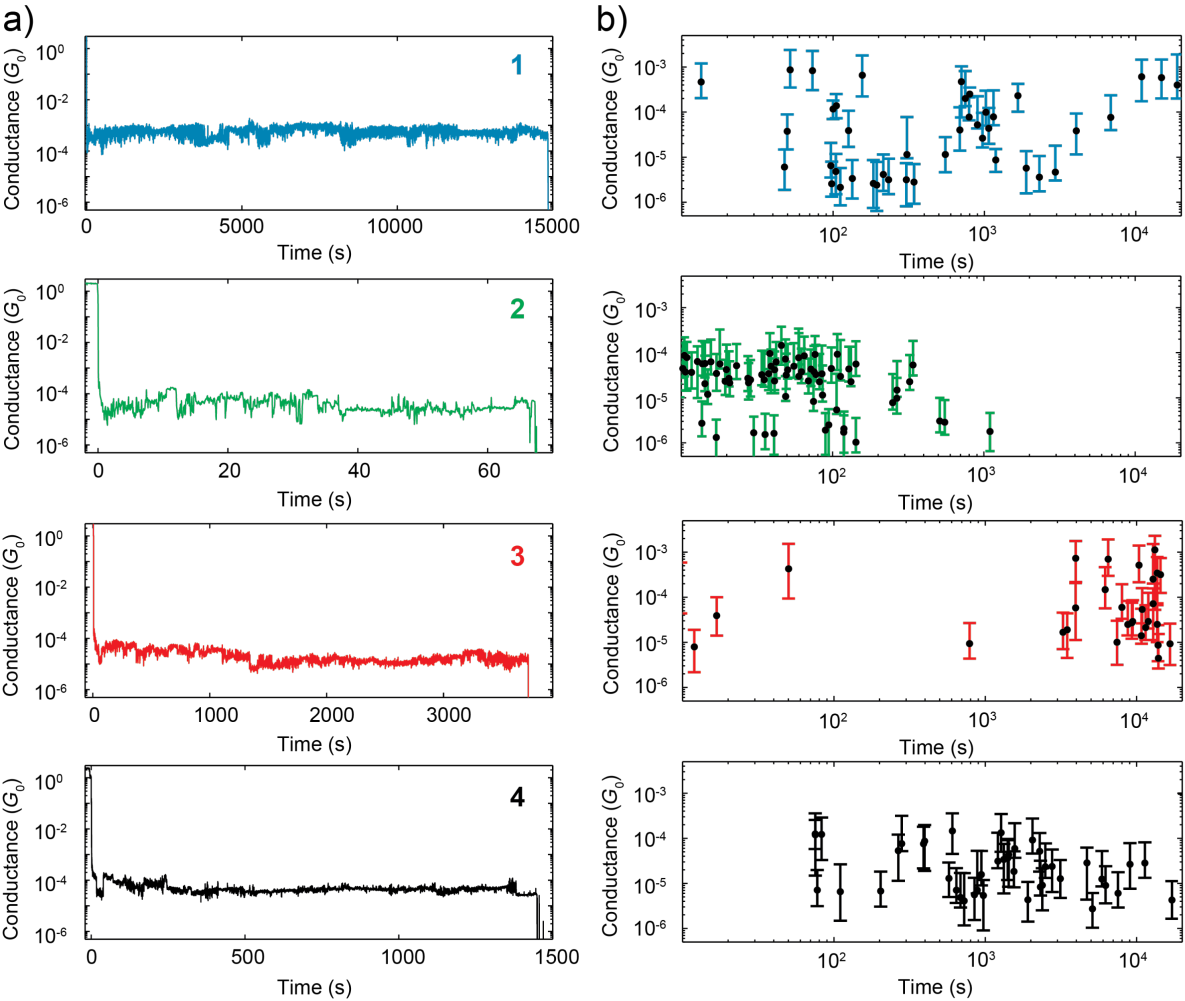
(a) Conductance–time traces measured for self-breaking junctions formed with molecules **1**–**4**. (b) Total time and conductance extracted from the time traces of molecules **1**–**4**; each dot represents a single conductance–time trace.

We now turn to the stability of the molecule-electrode interfaces and on how this mechanical stability depends on the anchoring groups. To this end, we have recorded conductance-time traces with the self-breaking technique [30–31,48–50]. Figure 7a shows four examples of conductance-time traces for the four different molecules. Remarkably, we find that the lifetime of the molecular junctions formed with the self-breaking method can reach thousands of seconds. In contrast the lifetime of empty junctions, measured with and without the solvent, is a few seconds at most, as shown in Figure S2 of the Supporting Information File 1.

In order to quantify the mechanical stability we extract from each individual time trace the lifetime, defined as the time it takes for the junction to break from 10^−1^*G*_0_ to 10^−6.5^*G*_0_, and the spread in conductance, defined as the variance of the distribution of the conductance values of each individual trace. The results from this analysis are plotted in Figure 7b; each dot represents the lifetime and the vertical bars the spanned conductance range. The different patterns observed for the four molecules are a first indication that the four anchoring groups exhibit different mechanical properties and that the breaking of the junctions is determined by the interface between the molecule and the gold and not by the gold stability alone.

Inspection of Figure 7b shows that thiol groups form junctions characterized by the largest spread in lifetime and conductance. Methyl sulfide, pyridine and amine anchoring groups show less spread in the lifetime of the junctions. The lifetime follows the trend Py *>* SAc 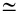 NH_2_*>* SMe. Interestingly, the conductance values of SAc and Py, in the self-breaking experiment, are different from the values found in the fast breaking and *I*–*V* measurements. The junctions formed with Py have a conductance one to two orders of magnitude higher, while SAc shows an order of magnitude decrease in conductance, in about half of the cases. On the other hand NH_2_ and SMe show the same conductance in all the experiments. An explanation for this behavior may be related to variations in the potential landscapes experienced by the molecules while stretching and its variability in the different types of experiments because of the different time-scales involved. More specifically, the potential landscape of the gold/molecule interfaces may contain different minima in space, that can give rise to different conductance values for the same molecule. When measuring in the self-breaking regime, it is easier for the molecule-metal system to explore a larger fraction of the configuration space than when measuring in the fast-breaking regime, giving rise to more variability in the probed conductance of the self-breaking experiment.

**Figure 8:**
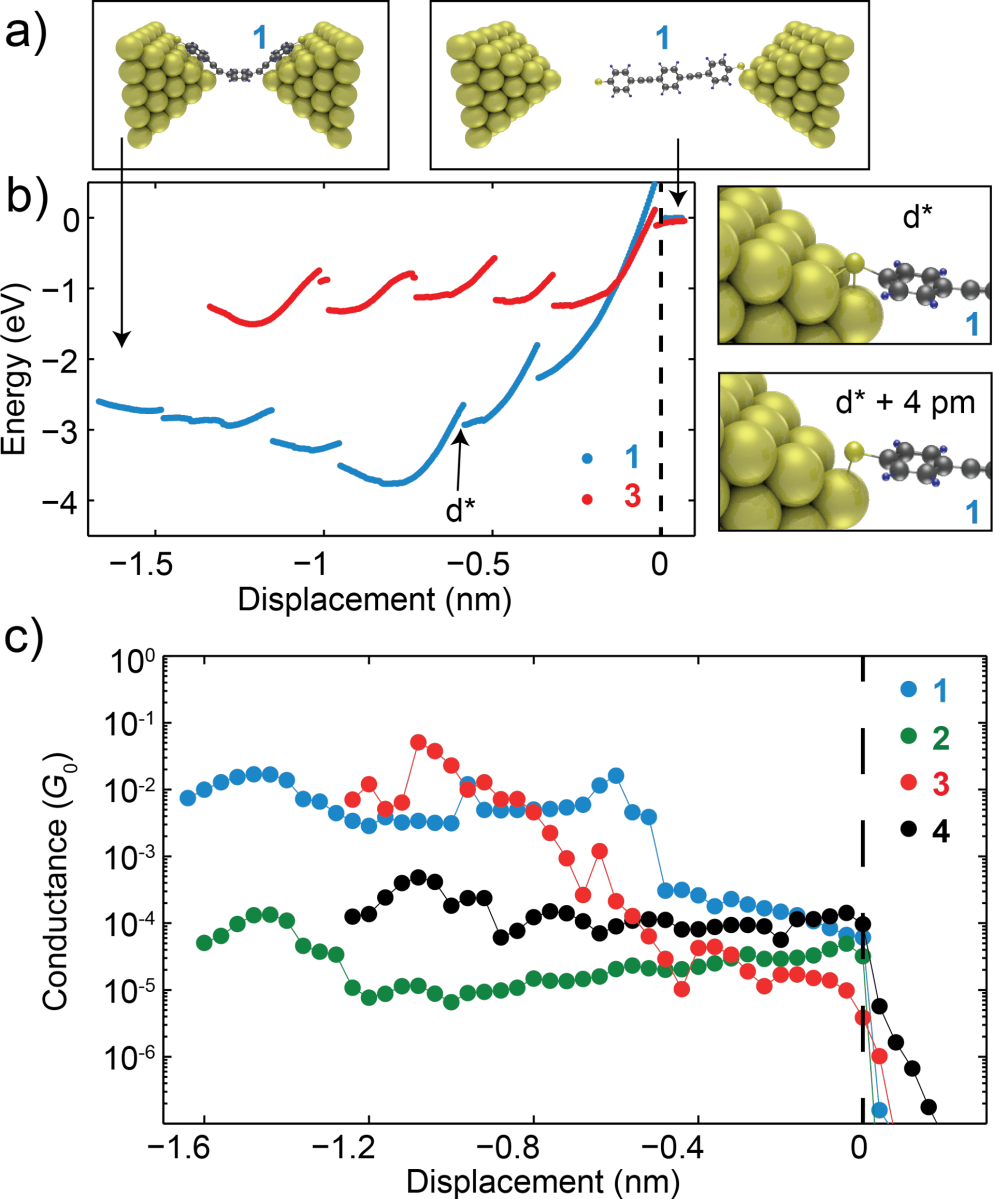
(a) Geometry of the molecular junction **1** at two different positions along the stretching. (b) Binding energy computed from DFT for molecules **1** and **3** as a function of electrodes distance. (c) Conductance of molecules **1**–**4** computed with NEGF at various positions while stretching the junctions.

To gain more insight in the different anchoring geometry and the observed variations in lifetimes and conductance values, we have performed DFT calculations by modelling the molecule of interest in between two pyramidal gold electrodes as described in the methods section. We start from an initial gap between the electrodes of 0.7 nm with a molecule bridging in between as shown in Figure 8a in the case of molecule **1**. Subsequently, we separate the electrodes in steps of 4 pm, we relax the geometry and perform a single-point calculation at each position, until we reach the rupture of the molecular junction, shown in the case of **1** in the right panel of Figure 8a. We have repeated the same stretching calculations by including dispersion correction in the Grimme dispersion corrected PBE implementation DFT-D3-BJ [51]. A comparison between the calculations with and without dispersion correction is shown in Section 6 of Supporting Information File 1.

Figure 8b plots the binding energy of the junctions formed with molecules **1** and **3** while stretching. Looking at the shape of the energy versus distance traces one can recognize continuous bowl-shaped segments separated by abrupt jumps. These jumps correspond to atomic rearrangements, typically at the gold-molecule interfaces as shown in Figure 8b for molecule **1** for the jump observed at position d^*^. When stretching the junction with molecule **1** the energy first decreases, so that the molecule finds an energetically more favorable configuration, and then increases. Molecule **3**, on the other hand, has a flatter profile for the energy, suggesting that the molecule–electrode interaction is less dependent on the position. The binding energy of junctions with **1** and **3** is larger than 1 eV for most of the positions, in agreement with literature [20,52]. This indicates that the direct thermal breaking of the gold-anchoring group bonds at room temperature is unlikely, since the thermal energy available is *k*_B_*T* = 25 meV. Interestingly, both SAc and Py can form long living junctions in the self-breaking experiment (lifetime larger than 10000 s).

At different positions separated by 40 pm, along the stretching curves, we have also calculated the transmission through the junction. By extracting the value of the transmission at the Fermi energy we reconstruct conductance versus displacement traces. Figure 8c shows the computed conductance versus displacement for all four molecular junctions. One can directly notice that, during the stretching of MeS and NH_2_ junctions, the conductance remains almost constant and transport in this case is rather insensitive to the junction configuration. This observation can explain the consistency of the conductance values measured on MeS and NH_2_ junctions formed with the three different techniques (fast-breaking, *I*–*V*s, self-breaking). The computed conductance-displacement curves of SAc and Py, on the other hand, show a larger variation. In particular Py shows a slanted region, where the conductance decreases by three orders of magnitude in the first 0.8 nm, and a plateau in the last 0.4 nm of stretching. The calculations of molecule **1** with the SAc anchoring groups show two plateaus, one at about 10^−2^*G*_0_ in the first 1 nm of stretching and the other at 2 · 10^−4^*G*_0_ in the last 0.5 nm of stretching. In the fast breaking experiments we observe that most of the SAc junctions show a conductance centered around 10^−4^*G*_0_. In the slow-breaking experiment we apparently also access to configurations with a lower conductance. We finally notice that our results are in broad agreement with previous studies from Wandlowski’s group [20,23].

## Conclusion

We have compared four different aurophilic anchoring groups widely used in molecular-scale electronics and investigated their influence, on the electrical and mechanical properties of single-molecule junctions, formed with MCBJ gold nano-electrodes. From the conductance breaking traces we find that the four different anchoring groups allow for the formation of single-molecule junctions and that the low-bias conductance of the junctions follows the trend: SAc *>* SMe 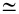 NH_2_*>* Py. A single-level model analysis of the *I*–*V*s shows that SAc groups give the best electronic coupling and level alignment to the gold nano-electrodes. To investigate the stability and the mechanical properties of single-molecule junctions, self-breaking measurements have been performed. We find that the lifetime, a measure for the mechanical stability, depends strongly on the anchoring group. Py anchoring groups give the largest lifetime suggesting a high stability of the molecule–metal bond that can involve the interaction of the full pyridyl rings with the gold. A larger spread in conductance, in respect to fast breaking measurements, suggest that in the self-breaking regime a larger part of the configuration space is accessible. DFT and NEGF calculations corroborate the observed trends in the experiments; more detailed calculations would be helpful to further characterize the metal-anchoring group dynamics and its influence on the mechanical and electronic properties.

## Supporting Information

File 1 Supporting Information features the synthesis of molecules **2**–**4**, the characterization of a bare gold sample where no molecules were deposited, additional details about DFT calculations, the analysis of the fitting parameters of the *I*–*V* s of molecules **1**–**4**.
