# Supplementary material for: Electrical properties and mechanical stability of anchoring groups for single-molecule electronics
Source: Beilstein J Nanotechnol. 2015 Jul 17;6:1558–67. doi: 10.3762/bjnano.6.159 (PMC4578406; doi:10.3762/bjnano.6.159)
Supplement: File 1 — Supporting Information features the synthesis of molecules 2–4, the characterization of a bare gold sample where no molecules were deposited, additional details about DFT calculations, the analysis of the fitting parameters of the I–V s of molecules 1–4. [file Beilstein_J_Nanotechnol-06-1558-s001.pdf]

Supporting Information

for

Electrical properties and mechanical stability of anchoring groups for single-molecule electronics

Riccardo Frisenda[1], Simge Tarkuç[2,3], Elena Galán[2], Mickael L. Perrin[1], Rienk Eelkema[2], Ferdinand C. Grozema[2] and Herre S. J. van der Zant[*1]

Address: [1]Kavli Institute of Nanoscience, Delft University of Technology, Lorentzweg 1, 2628 CJ Delft, The Netherlands, [2]Opto-electronic Materials and Advanced Soft Matter, Department of Chemical Engineering, Delft University of Technology,Julianalaan 136, 2628 BL Delft, The Netherlands and [3]Current address: Arcelik A.S.Central R&D Department, 34950 Tuzla/Istanbul, Turkey

Email: Herre S. J. van der Zant - h.s.j.vanderzant@tudelft.nl

* Corresponding author

Experimental part

1. Measurements and general methods

1.1. General characterization methods

^{1}H NMR and ^{13}C NMR spectra were performed in the appropriate deuterated solvents with tetramethylsilane as internal standard on a Bruker Advance spectrometer at 400 MHz (^{1}H) and 100 MHz (^{13}C); chemical shifts (δ) are reported in parts per million.

1.2. Synthesis and characterization

All starting materials were purchased from Sigma-Aldrich, Acros and Alfa Aesar and used as received without further purification. Analytical thin layer chromatography (TLC) was performed on silica gel 60-F254 (Merck) plates and detected under UV lamp. Column chromatography was performed on silica gel 60 (Aldrich). S,S′-[1,4-phenylenebis(2,1-ethynediyl-4,1-phenylene)]bis(thioacetate) (**1**) was purchased from Aldrich Chemical Co. and used as recieved.

1.2.1. Synthesis of 1,4-bis((4-(methylthio)phenyl)ethynyl)benzene (2)

The synthesis of 1,4-bis((4-(methylthio)phenyl)ethynyl)benzene (**2**) was achieved by means of palladium-mediated cross-coupling reactions of 1-bromo-4-methylthiobenzene (**b**) with 1,4-bis((trimethylsilyl)ethynyl)benzene (**c**) as shown in Scheme S1.

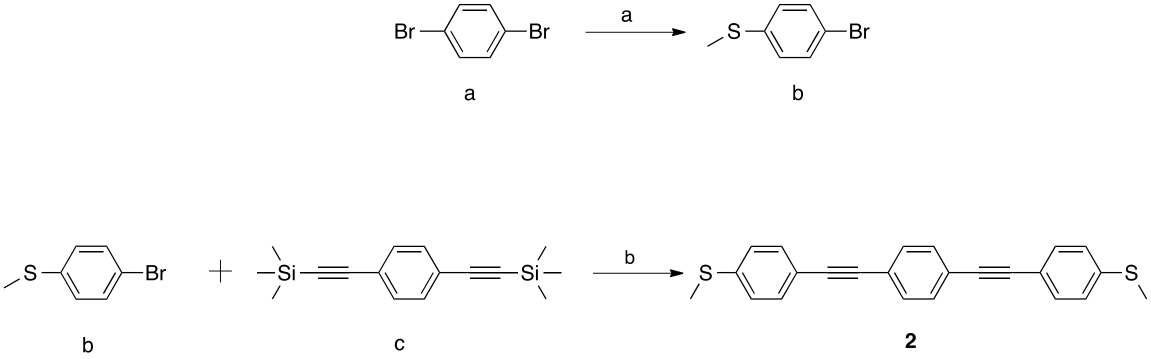

Scheme S1: Synthesis of 1,4-bis((4-(methylthio)phenyl)ethynyl)benzene (**2**). Reagents and conditions: a) n-BuLi, MeSSMe, Et_2O; b) $Pd(PPh_3)_2Cl_2$, CuI, DBU, THF, H_2O.

1-bromo-4-methylthiobenzene (b) [S1]. n-BuLi (2.5 M in hexane, 4.6 mL, 0.01 mol) was added dropwise to a stirred solution of 1,4-dibromobenzene (2.5 g, 0.01 mol) in dry Et_2O (20 mL) at -78 °C. After stirring at -78 °C for 45 min under Argon, dimethyldisulfide (1.05 mL, 0.01 mol) in dry Et_2O (4 mL) was added. The reaction mixture was stirred at -78 °C for 2 h and allowed warm to -35 °C over 2 h. After that the reaction mixture was poured into a mixture of NH_4Cl in ice. The organic phase was separated, the aqueous phase was re-extracted with Et_2O, and the combined organic phase was washed with saturated solution of NaCl, dried over $MgSO_4$. The solvent was removed under vacuum. The crude product was purified by silica gel column chromatography (petroleum ether) to afford 1.4g (6.9 mmol, 63%) of 1-bromo-4-methylthiobenzene (**2**): ^1H NMR ($CDCl_3$, 400 MHz): δ 7.39(d, 2H, J = 8.6 Hz), 7.11 (d, 2H, J = 8.6 Hz), 2.46 (s, 3H). ^{13}C NMR ($CDCl_3$, 100 MHz): δ 137.5, 131.6, 127.9, 118.4, 15.7.

1,4-bis((4-(methylthio)phenyl)ethynyl)benzene (2) [S2]**.**

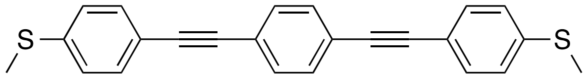

2

To a solution of $Pd(PPh_3)Cl_2$ (62 mg, 0.08 mmol) and CuI (28 mg, 0.04 mmol) in dry THF (30 mL) was added 1-bromo-4-methylthiobenzene (**b**) (300 mg, 1.48 mmol) at room temperature. After the system was purged with argon, 1,8-diazabicyclo[5.4.0]undec-7-ene (DBU) (1.35 g, 8.86 mmol) was added by syringe.

Argon-sparged 1,4-bis((trimethylsilyl)ethynyl)benzene (**c**) (200 mg, 0.80 mmol) was injected into the reaction flask and followed immediately by distilled water (0.01 mL, 0.591 mmol). The reaction mixture was stirred at reflux for 18 h. All volatile material was removed under vacuum and the residue was dissolved in CH_2Cl_2 (50 mL). The organic layer was washed with saturated aqueous NaCl (2 x 50 mL), dried over $MgSO_4$ and the solvent removed under vacuum. The crude product was purified by silica gel column chromatography (dichloromethane: petroleum ether, 1:2 (V:V)) to afford 109 mg (0.29 mmol, 40%) of 1,4-bis((4-(methylthio)phenyl)ethynyl)benzene (**2**) as a white solid. ^{1}H NMR ($CDCl_3$, 400 MHz):δ 7.49(s, 4H), 7.44 (d, 4H, *J* = 8.3 Hz), 7.21 (d, 4H, *J* = 8.3 Hz), 2.51 (s, 6H). ^{13}C NMR ($CDCl_3$, 100 MHz):δ 131.9, 131.9, 131.5, 131.4, 125.8, 125.8, 100.0, 89.2,15.3. EI-MS: *m/z* calcd for $C_{24}H_{18}S_2$, 370.08; found 370.10.

1.2.2. Synthesis of 1,4-bis(pyridine-4-ylethynyl)benzene (3) [S3].

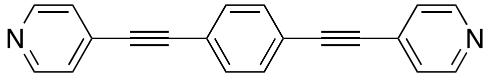

3

1,4-bis(pyridine-4-ylethynyl)benzene **(3)** was synthesized according to the method described in the literature [S3]. ^{1}H NMR ($CDCl_3$, 400 MHz): δ 8.62 (d, 4H, *J* = 5.6 Hz), 7.56 (s, 4H), 7.39 (d, 4H, *J* = 5.8 Hz). ^{13}C NMR ($CDCl_3$, 100 MHz): δ 149.3, 132.0, 125.7, 122.8, 100.0, 88.7. EIMS: *m/z* calcd for $C_{20}H_{12}N_2$, 280.10; found 280.10.

1.2.3. Synthesis of 1,4-bis((4'-aminophenyl)ethynyl)benzene (4) [S4].

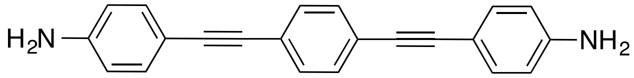

4

1,4-bis((4'-aminophenyl)ethynyl)benzene **(4)** was synthesized according to the method described in the literature [S4]. ^{1}H NMR (DMSO-d_6, 400 MHz): δ 7.43 (s, 4H), 7.20 (d, 4H, *J* = 8.5 Hz), 6.56 (d, 4H, *J* = 8.5 Hz), 5.62 (s, 4H). ^{13}C NMR (DMSO-d_6, 100 MHz): δ 149.6, 132.5, 130.8, 122.4, 113.5, 107.7, 93.1, 86.3. EIMS: *m/z* calcd for $C_{22}H_{16}N_2$, 308.13; found 308.15.

2. MCBJ sample electrical characterization

2.1 Breaking traces and conductance histograms of control samples

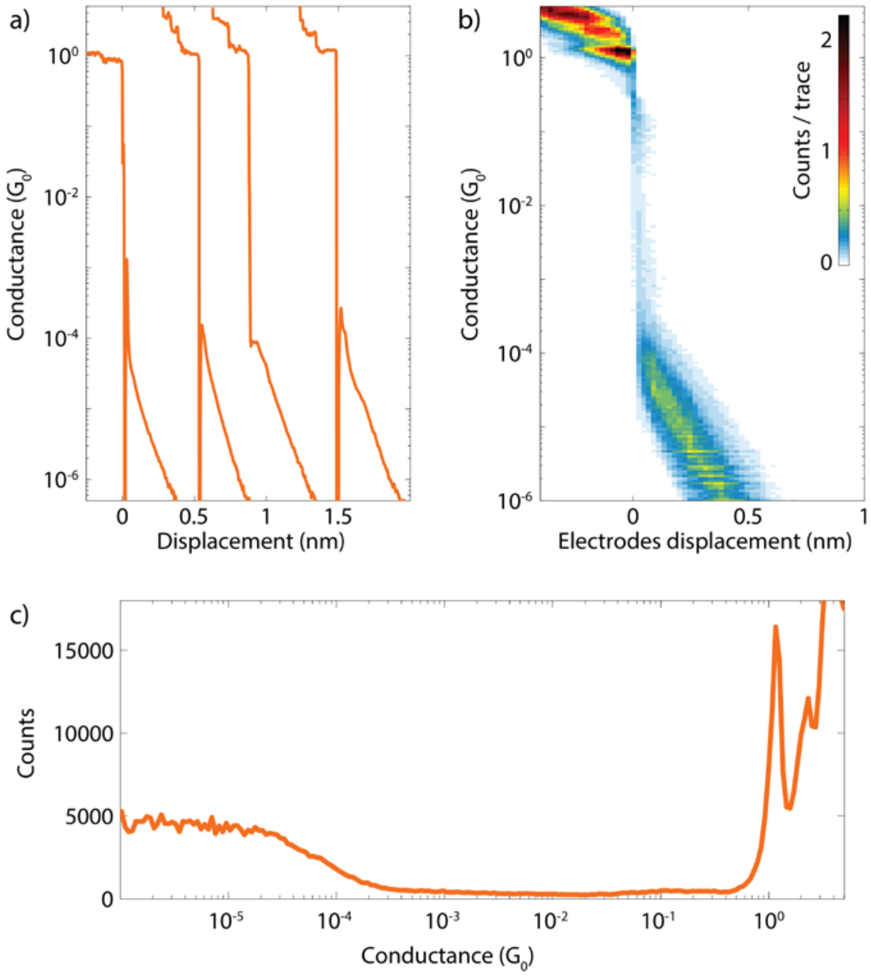

Figure S1: a) Examples of individual breaking traces for junctions in ambient conditions. The bias is 0.1 V and the electrodes separation speed is 5 nm/s. b) Conductance versus displacement histogram obtained from 2200 breaking traces. c) Conductance histogram obtained by summing the histogram in b) along the displacement axis. The conductance is logarithmically binned with 30 bins/decade and the displacement is linearly binned with 45 bins/nm.

2.2 Self breaking of a gold wire

We performed self-breaking measurements on a bare gold sample, using the same technique as described in the main text and shown in Figure 5 for molecules **1**-**4**. Figure S2a shows five examples of time traces measured on bare gold in the self-breaking regime, without driving the electrodes. Figure S2b reports the conductance histogram built from 70 of such conductance-time traces. Figure S2c shows the extracted conductance and lifetime of the bare gold junctions. These plots indicate that a bare gold sample has on average a shorter lifetime than a molecular junction, when measured in the self-breaking regime. The largest lifetime observed for a gold junction is less than 30 seconds while for junctions formed with molecules **1**-**4** the lifetime can exceed 10000 seconds in some cases.

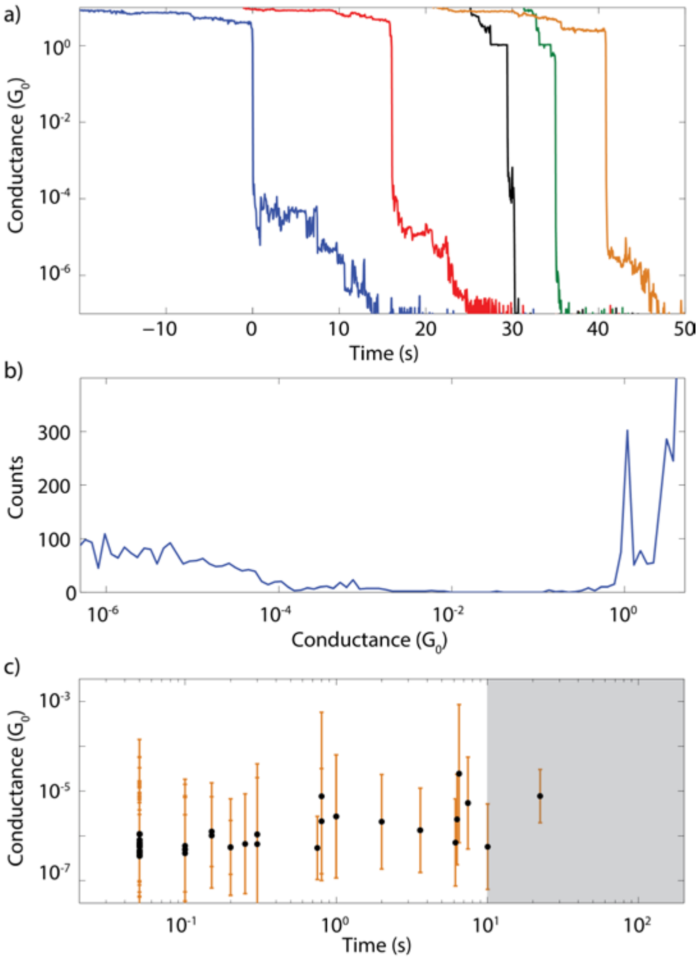

Figure S2: a) Examples of individual conductance versus time traces for junctions exposed to air in the self-breaking regime. The bias is 0.1 V. b) Conductance histogram built from 70 self-breaking traces. c) Total time and conductance extracted from the time traces of a sample exposed to air. Each dot represents a single conductance-time trace. The shaded area indicates the region covered by conductance-time plots in Figure 5b of the main text.

3. Fit of Breit-Wigner parameters distributions

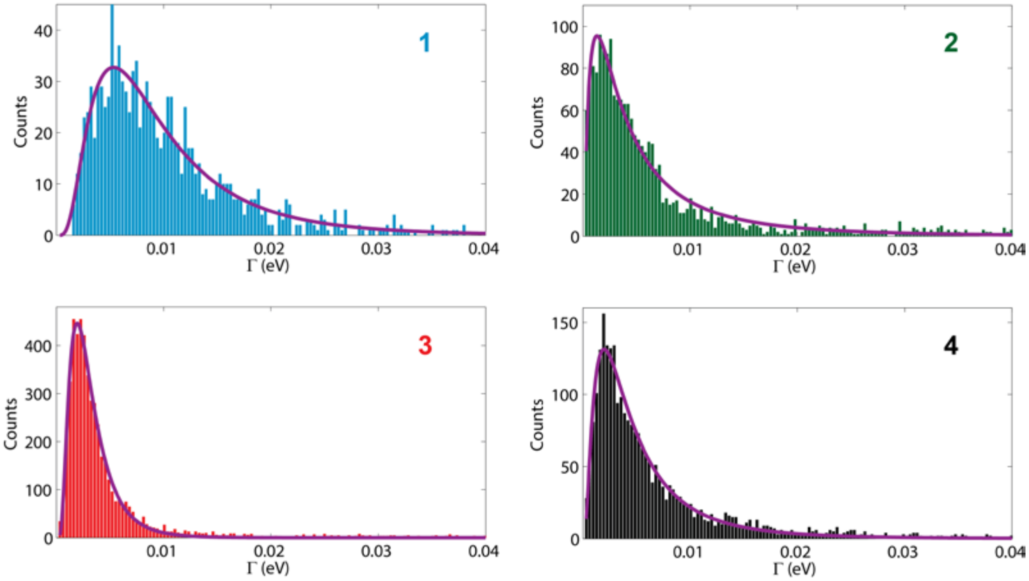

Figure S3: Histograms of the electronic coupling extracted by fitting the I-Vs of molecules **1**-**4** to the single-level model. The red lines are fits to log-normal distributions; the fitting parameters are given in Table S1.

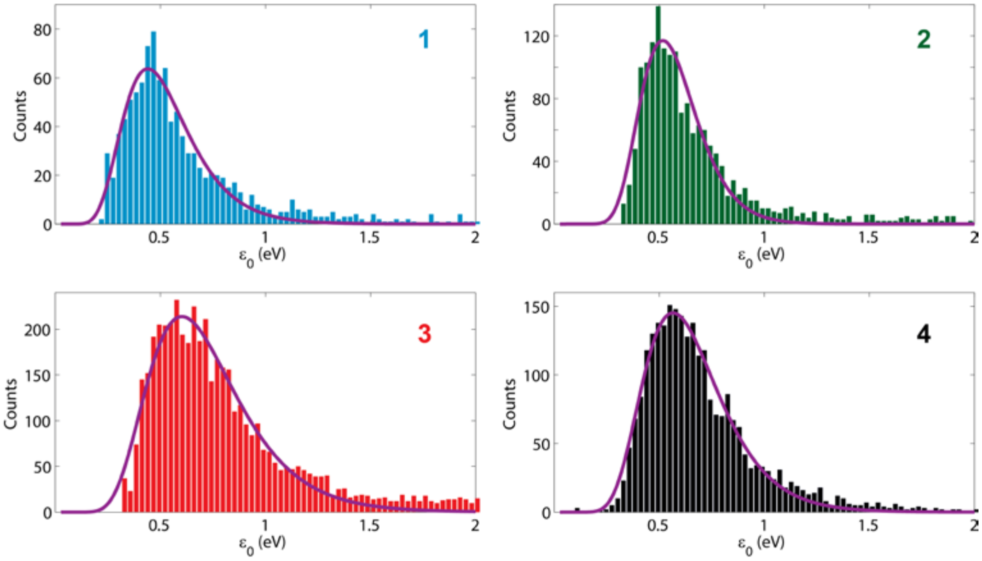

Figure S4: Histograms of the level alignment extracted by fitting the I-Vs of molecules **1**-**4** to the single-level model. The red lines are fits to log-normal distributions; the fitting parameters are given in Table S2.

Table S1: Fitting parameters of the electronic coupling distributions reported in Figure S3.

	σ	μ
**1 (SH)**	1.49	-4.78
**2 (SMe)**	0.93	-5.46
**3 (Py)**	1.17	-5.49
**4 (NH_2)**	1.68	-5.88

Table S2: Fitting parameters of the level alignment distributions reported in Figure S4.

	σ	μ
**1 (SH)**	2.86	-0.70
**2 (SMe)**	3.88	-0.59
**3 (Py)**	2.86	-0.38
**4 (NH_2)**	3.15	-0.47

4. Conductance histograms of self-breaking measurements

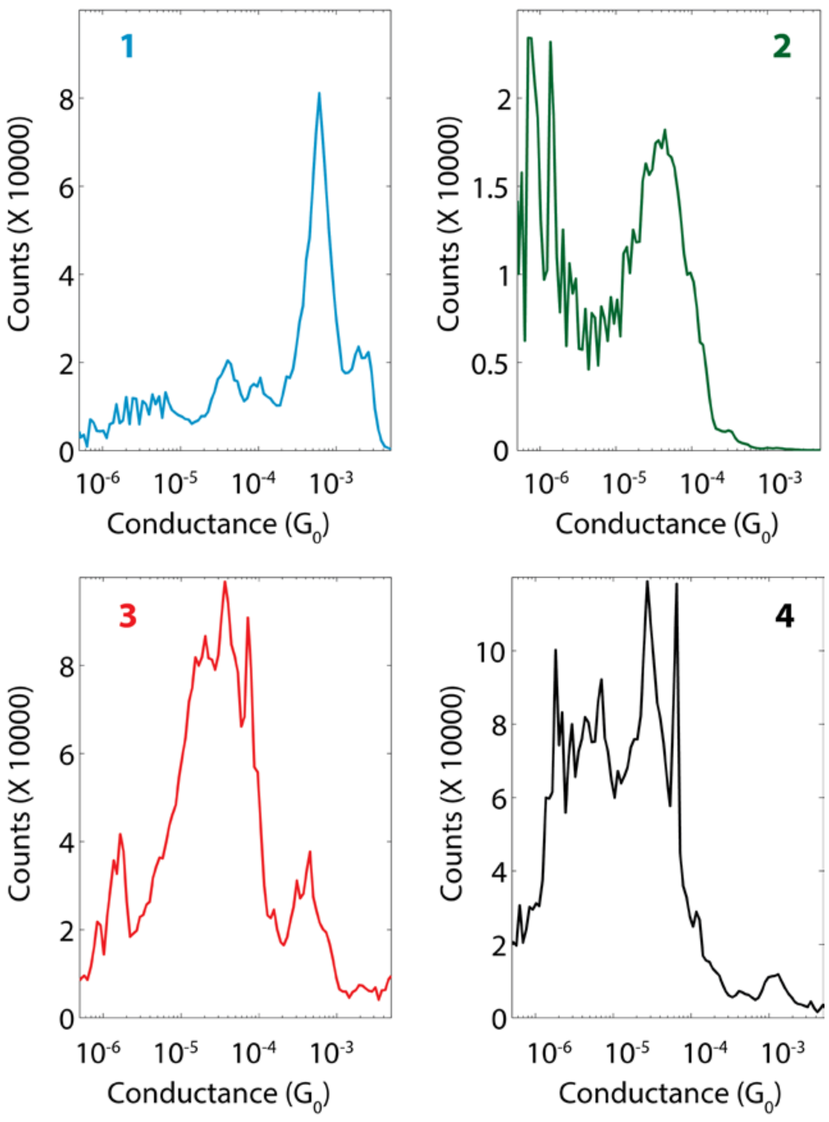

Figure S5: Conductance histogram built from the conductance-time self-breaking traces of molecules **1-4** data reported in Figure 5 of the main text.

5. DFT calculated transmission

Figure S6 shows the transmission calculated from DFT for junctions with molecules **1**-**4** as solid lines. The geometries are shown in Figure S7, with the molecules fully extended between the electrodes. The transmission of each molecule, calculated by applying DFT+Σ corrections, is shifted along the energy axis in order to match the level alignment found in the experimental I-Vs. Table S3 summarizes the Fermi energy shift in the four cases. The two-dimensional colour-maps in Figure S6 represent the transmission values explored in the experimental I-Vs. For each I-V, fitted to the Breit-Wigner single level model, we calculate the Lorentzian transmission defined in Equation 2 of the main text, using the fitting parameters to the I-V. We then construct two-dimensional histograms of all the Lorentzian transmission calculated in this way.

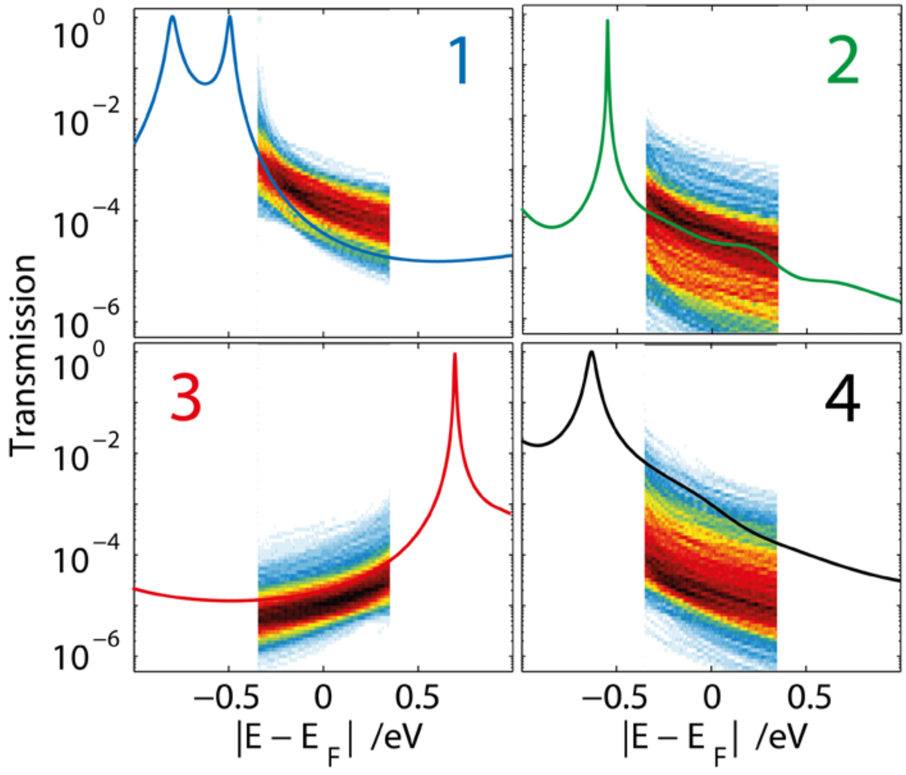

Figure S6: Transmission of molecules **1**-**4** calculated from DFT (solid lines). The color-maps in the background, built from the individual I-Vs measured on molecules **1**-**4**, represent the transmission values explored in the experiments.

Table S3: Single-level model parameters extracted from the DFT transmissions.

	$\Delta E_F{}^a$ (eV)	$\Gamma_{(DFT)}$ (meV)	$G_{(DFT)}{}^b$ (G_0)
1 (SH)	0.24	9.6	$6.1 \cdot 10^{-5}$
2 (SMe)	-1.02	1.8	$3.2 \cdot 10^{-5}$
3 (Py)	-0.49	3.2	$1.4 \cdot 10^{-5}$
4 (NH_2)	-0.73	13.2	$5.7 \cdot 10^{-4}$

[a] Shift of the Fermi energy (from the value of found for gold E_F = -4.9 eV), in the DFT calculated transmission, in order to match the level alignment found in the experiments; [b] Conductance at the Fermi energy.

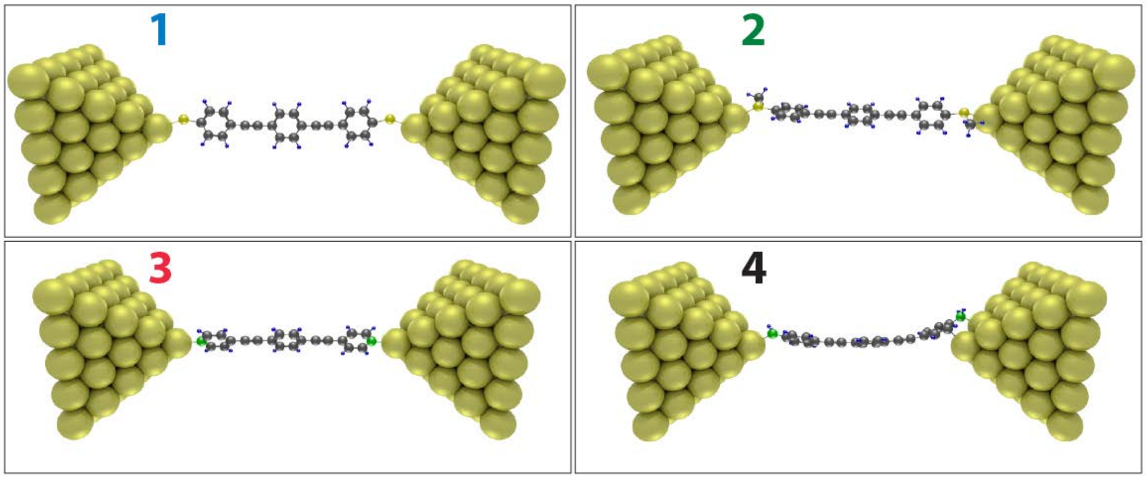

Figure S7: Junction geometries for the transmission calculations in Figure S6. These geometries correspond to fully extended molecules in the junctions just prior to the final rupture.

6. DFT-D3 dispersion corrected energy calculations

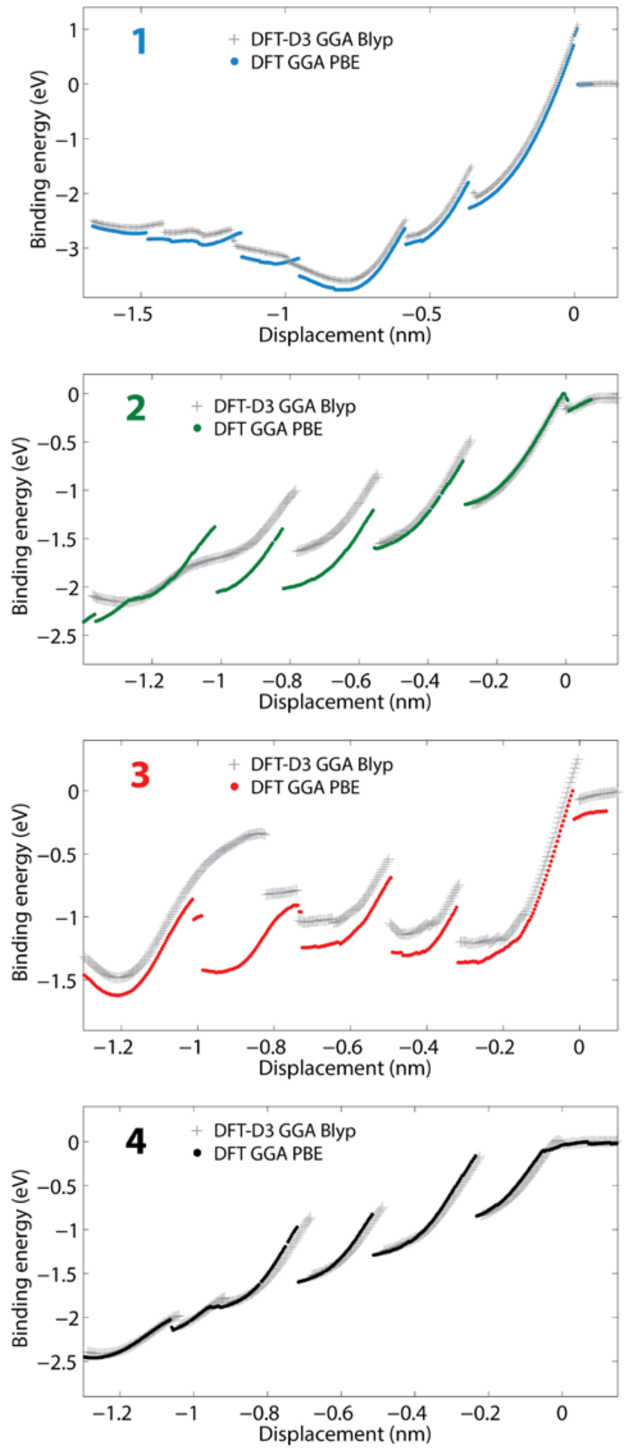

Figure S9: Binding energy versus electrodes displacement calculated from DFT with (coloured circles) and without (grey crosses) dispersion corrections for molecules **1**-**4**.

References

S1. Isabel C. González, Leon N. Davis and Charles K. Smith, II, *Bioorganic & Medicinal Chemistry Letters* 14 (2004) 4037–4043. doi:10.1016/j.bmcl.2004.05.044

S2. Matthew J. Mio, Lucas C. Kopel, Julia B. Braun, Tendai L. Gadzikwa, Kami L. Hull, Ronald G. Brisbois, Christopher J. Markworth, and Paul A. Grieco, *Organic Letters* 4 (2002) 3199-3202. doi:10.1021/ol026266n

S3. Neil. R. Champness, Andrei N. Khlobystov, Alexander G. Majuga, Martin Schröder and Nikolai V. Zyk, *Tetrahedron Letters* 40 (1999) 5413-5416. doi:10.1016/S0040-4039(99)01019-9

S4. Qi Lu, Ke Liu, Hongming Zhang, Zhibo Du, Xianhong Wang and Fosong Wang, *ACS Nano* 3 (12), (2009) 3861-3868. doi 10.1021/nn9012687
